# Low back pain among school teachers in Botswana, prevalence and risk factors

**DOI:** 10.1186/1471-2474-15-359

**Published:** 2014-10-30

**Authors:** Patience N Erick, Derek R Smith

**Affiliations:** School of Health Sciences, Faculty of Health and Medicine, University of Newcastle, 10 Chittaway Road, Ourimbah, New South Wales 2258 Australia

## Abstract

**Background:**

Although low back pain (LBP) represents a common occupational problem, few epidemiological studies have investigated the prevalence and risk factors for LBP among school teachers, particularly in Africa. School teachers are known to represent an occupational group among which there appears to be a high prevalence of LBP. The objective of this study was, therefore, to conduct one of the first epidemiological investigations of LBP among teachers in Botswana.

**Methods:**

A cross-sectional study was conducted among teachers in Botswana using self-administered questionnaires which were distributed to 3100 randomly selected school teachers and collected over a five-month period between July and November 2012. The questionnaire included low back pain information, demographic data, lifestyle, work-related characteristics and psychosocial factors. Data were analysed using Chi-squared and logistic regression models. The 12 month prevalence and LBP disability and associated risk factors were also analysed.

**Results:**

A total of 1747 teachers returned completed questionnaires, yielding a response rate of 56.3%. The 12-month prevalence of LBP was 55.7%, with 67.1% of them reporting minimal disability. The results of logistic regression analysis revealed that female gender [OR: 1.51, 95% CI: 1.14-2.00] and previous back injury [OR: 9.67, 95% CI: 4.94-18.93] were positively correlated to LBP. Awkward arm position [OR: 1.81, 95% CI: 1.24-2.62] and high psychological job demands [OR: 1.40, 95% CI: 1.02-1.93] were also significantly associated with LBP. Regular physical exercise was negatively associated with LBP [OR: 0.63, 95% CI: 0.43-0.93]. Female gender [OR: 2.67, 95% CI: 1.52-3.99] and previous back injury [OR: 3.01, 95% CI: 1.92-4.74] were also positively associated with LBP disability.

**Conclusion:**

The prevalence of LBP appears to be high among school teachers in Botswana. A wide variety of LBP risk factors were identified in this study. Female gender and previous injury were both associated with LBP presence and disability. The complex nature of LBP risk factors found in this study suggests than no single specific preventative or intervention strategy will help in reducing these conditions. As such, to help reduce the prevalence, progression and burden of LBP among Botswana teachers, a greater emphasis should now be placed on ergonomics education, regular physical exercise and occupational stress.

**Electronic supplementary material:**

The online version of this article (doi:10.1186/1471-2474-15-359) contains supplementary material, which is available to authorized users.

## Background

Low back pain (LBP) is widely acknowledged as an important health and socio-economic problem which plagues a large segment of the population in industrialised countries[1]. The situation is reportedly even worse in developing countries, with suboptimal working conditions in many industries and an acute lack of awareness of ergonomics issues, education and training programmes[2]. LBP does not only lead to a poorer quality of life for individuals, but also decreased labour productivity due to time off work, increased absenteeism and early retirement. Moreover, LBP is also associated with escalating medical costs[1]. This condition often occurs as a result of cumulative trauma and can affect the bones, muscles and their attachments, as well as nerves and blood supply[3]. Considerable focus has been on back injuries and musculoskeletal disorders of workers in health care[3–5] and other industries[2, 6]. However, a significant body of research has also recently suggested that school teachers are at an increased risk of musculoskeletal disorders[7], with prevalence rates reported at between 12% and 84%[8, 9].

Work-related tasks are widely considered to be a major cause of LBP among teachers. It is postulated that awkward posture, prolonged sitting when working on students’ work and when preparing for lessons[10], and inappropriate furniture[11] are contributing factors for LBP among teachers. An increasing body of research has demonstrated important links between not only physical demands of one’s job, but also the psychosocial and structural factors that influence workers’ lives at work[4, 12–14]. Despite these facts, there are few studies examining which of the wide spectrum of risk factors are predictive of LBP in the teaching profession. It is important in policy making to investigate factors that relate to LBP among teachers and thereafter take measures to prevent such conditions so as to protect teachers’ health and the quality of education that their students receive.

Despite their large demographic and associated potential for occupational health problems, few epidemiological studies have investigated LBP prevalence and risk factors among teachers. Hence the aim of this study was to analyse the prevalence and distribution of LBP among teachers in Botswana and to establish risk factors that influence the development and the extent of their symptoms.

## Methods

### Location and background

A large cross-sectional study of musculoskeletal disorders was conducted among teachers in Botswana between July 2012 and November 2012. From ten education regions in the country, seven regions were selected in order for the study to be representative of all teachers in Botswana and also it was not have been feasible to sample all education regions. The regions were taken as clusters and numbered one to 10. Using a random sequence generator, the seven first regions were selected. From these randomly selected regions, schools were stratified into primary or secondary schools and alphabetically compiled into two different lists. There was no national data available to show how many school teachers were in each region. As such, questionnaires were equally distributed to all regions that formed part of the study. In 2010, there were 11711 primary and 13173 secondary school teachers employed by the government of Botswana through Department of Public Service Management (DPSM)[15]. Power calculations indicated that a sample size of 1537 each group would be required to calculate result at the 95% significance level. This number was then rounded up to 1550 for practical purposes. A total of 1550 primary and 1550 secondary teachers were invited to participate from randomly selected 107 primary and 57 secondary schools. All school teachers in those schools were invited to take part in the study. The number of teachers in schools varied from one school to the other depending on the level of the school and the number of students. In primary schools, for instance, one school can have about six teachers while another school can have 27 teachers and, in senior secondary schools, one school can have as many as 120 teachers. Permission to conduct the research in the selected schools was sought from school heads. Not all agreed to participate; however, and where a school head declined to participate, their school was then replaced by another from the randomization list. The study commenced after obtaining ethical clearance from University of Newcastle Human Research Ethics Committee and a research permit from Ministry of Education and Skills Development in Botswana (MoESD). Postal questionnaires were used to collect data from participants and informed consent was implied by voluntarily completing and returning the questionnaire. Teachers were also given information sheets describing the procedure and objectives of the study.

### Questionnaire design

An anonymous self-administered questionnaire was used to assess the demographic and individual data, low back pain, low back disability, and physical and psychosocial exposures during work among teachers. The five page questionnaire was divided into four sections with the first section covering demographic items such as gender, age, education level, marital status and tobacco smoking. The second section assessed participants’ low back complaints and previous low back injury using the Standardized Nordic Questionnaire (SNQ)[16]. Questions addressing the perceived level of low back disability constituted the third component and were adapted from Oswestry Disability Index (ODI)[17]. The last section of the questionnaire assessed psychosocial and physical work demands using the Job Content questionnaire (JCQ)[18]. To make the questionnaire easy to complete, it consisted of a number of tick-box style and anatomical diagram with shaded areas. The questionnaire was administered in English.

### Statistical analysis

All data were coded and entered into SPSS 20.0 and analysed. Independent t-test and Chi-squared test were used to analyse quantitative and categorical data, respectively. Basic statistical associations between demographic, physical and psychosocial variables were initially evaluated using Chi-squared tests. Risk factors were then evaluated using logistic regression and expressed as Odds Ratios (OR) with 95% Confidence Intervals (95% CI). LBP was used as the dependent variable, with demographic items, lifestyle, workplace, physical and psychosocial factors used as independent variables. Probability values below 0.05 were regarded as statistically significant throughout all analyses.

## Results

### Participant demographics

A total of 3100 questionnaires were distributed to teachers from whom 1747 were returned, yielding a response rate of 56.3%. Fifteen questionnaires were excluded from analysis because they were not completed, leaving 1732 respondents, and giving an overall coverage rate of 55.9%. Of these respondents, 1003 (57.9%) were primary school teachers, while 559 (32.3%) and 170 (9.8%) were junior and senior secondary school teachers, respectively. The participants comprised of a higher proportion of female (72.7%) than male teachers (27.3%). The majority of respondents had ≤10 years of teaching experience (48.4%): 68.9% taught in junior secondary and 42.9% in senior secondary; while 38.0% taught in primary school. Table 1 lists the participants’ main demographic characteristics.

**Table 1:** **Demographic, life style and work characteristics of primary (n = 1003), junior secondary (n = 559) and senior secondary teachers (n = 170) in Botswana**

Characteristics	Primary school teachers	Junior secondary school teachers	Senior secondary school teachers	Overall
	%	%	%	%
Gender				
Male	17.0	39.2	48.2	27.3
Female	83.0	60.8	51.8	72.7
Age (years)				
≤30	15.6	34.3	10.2	21.1
31-40	29.5	49.4	53.6	38.3
41-50	40.4	14.1	31.7	31.0
>50	14.5	2.2	5.4	9.6
Body mass index				
<18.5	5.0	7.1	3.7	5.6
18-24.9	35.7	51.9	43.7	42.1
25-29.9	29.3	25.1	34.8	28.5
≥30	30.0	15.9	17.8	23.9
Marital status				
Single	53.8	54.7	42.4	53.0
Married	38.7	42.8	50.6	41.2
Separated/divorced/widowed	7.5	2.5	7.1	5.8
Education level				
Certificate	9.2	0.2	0.6	5.4
Diploma	71.7	53.1	0.6	58.7
Bachelors’ degree	19.1	46.7	98.8	35.9
Number of children less than 6 years				
1	73.9	72.5	73.0	73.2
≥2	26.1	27.5	27.0	26.8
Hours of physical exercise per week (hours)				
≤5	89.5	84.3	84.6	87.2
>5	10.5	15.7	15.4	12.8
Length of employment (years)				
≤10	38.0	68.9	42.9	48.4
11-20	34.0	28.3	47.1	33.4
21-30	24.1	2.5	8.8	15.6
>30	3.9	0.4	1.2	2.5
Work hours per week (hours)				
40	88.8	85.7	83.5	87.3
>40	11.2	14.3	16.5	12.7
Number of students taught on average				
≤25	5.2	25.8	9.4	12.2
26-30	20.4	8.1	4.7	14.9
31-35	39.4	11.3	15.3	27.9
36-40	32.6	32.6	55.9	34.9
>40	2.4	22.4	14.7	10.0
Extracurricular activities				
No	22.6	45.8	55.9	33.4
Yes	77.4	54.2	44.1	66.6

As shown in Table 2, the results suggest that there was a significant difference in age distribution for males (M = 36.29, SD = 7.02) and females (M = 39.34, SD = 8.62), p < 0.001. Similarly, there was a significant difference in body mass index (BMI) distribution for males and females (24.75 ± 5.78 vs 27.55 ± 7.00). A higher proportion of the single teachers were male (58.7%), while 42.5% of female teachers were married. The majority of teachers had a diploma (58.7%), and most of the teachers with a bachelor’s degree were male (43.4%), compared to 33.0% of female teachers. Similarly, the majority of males (46.4%) taught in junior secondary schools, while a higher proportion of female teachers (66.0%) taught in primary schools.

**Table 2:** **Descriptive statistics of individual, life style and work characteristics among male and female teachers in Botswana**

Characteristics	Male (n = 472)	Female (n = 1260)	Total (n = 1732)	***P*** value
Age	36.29 ± 7.02	39.34 ± 9.02	38.50 ± 8.62	**<0.001**
Body mass index	24.75 ± 5.78	27.55 ± 7.00	26.65 ± 6.76	**<0.001**
Length of employment	10.14 ± 6.31	13.36 ± 8.82	12.48 ± 8.34	**<0.001**
Cigarettes/day	5.88 ± 4.78	2.80 ± 1.64	5.59 ± 4.68	0.163
Marital status				**0.004**
Single	58.7	50.9	53.0	
Married	37.5	42.5	41.2	
Separated/divorced/widowed	3.8	6.6	5.8	
Educational level				**<0.001**
Certificate	1.7	6.8	5.4	
Diploma	54.9	60.2	58.7	
Bachelor degree	43.4	33.0	35.9	
Number of children less than 6 years				0.210
1	70.3	74.9	73.2	
≥2	29.7	25.1	26.8	
Smoking				**<0.001**
Smokers	10.8	0.4	3.2	
Ex-smokers	13.6	2.1	5.3	
Never smoked	75.6	97.5	91.5	
Physical exercise per week >5 hours	18.1	10.4	12.8	**<0.001**
School level				**<0.001**
Primary school	36.2	66.0	57.9	
Junior secondary	46.4	27.0	32.3	
Senior secondary	17.4	7.0	9.8	
Work hours per week >40 hours	14.6	12.0	12.7	0.166
Number of students >40	11.4	9.5	10.0	**<0.001**
Involved in extracurricular activities	69.9	65.4	66.6	0.086

P values were derived from either independent t-test for quantitative data or chi-squared test for categorical data. Statistically significant differences (p<0.05) are marked in bold.

A higher proportion of male teachers (18.1%) reported doing physical exercise for more than five hours a week, compared with females (10.4%). In addition, 11.4% of males taught more than 40 students in class, compared with females (9.5%). These findings were statistically significant. Similarly, a higher proportion of male teachers reported being involved in extracurricular activities when compared to female teachers. However, this finding was not statistically significant. There were no statistically significant differences between gender and having children less than 6 years and working for more than 40 hours a week.

### LBP prevalence

The 12-month self-reported prevalence of LBP among Botswana teachers was 55.7%. As shown in Table 3, female teachers had a significantly higher prevalence rate when compared to males (58.7% vs 47.7%, p < 0.001). Results demonstrated that teachers with previous back injury had the highest prevalence of LBP. There was a significant difference between teachers with and without previous injury in the prevalence of LBP (p < 0.001). Teachers who reported doing physical exercise ≤5 hours per week had the highest prevalence of LBP, compared to those who had more than 5 hours of physical exercise per week. Similarly, there were significant differences between hours of physical exercise in the prevalence of LBP (p = 0.024).

**Table 3:** **The 12 month prevalence of LBP among Botswana teachers in relation to individual and lifestyle factors**

Risk factors^a^	% with LBP	***P*** value
Gender		**<0.001**
Male	47.7	
Female	58.7	
Age (years)		0.356
≤30	53.5	
31-40	54.5	
41-50	58.9	
>50	56.4	
Body mass index		0.673
<18.5	50.7	
18-24.9	54.1	
25-29.9	56.5	
≥30	57.2	
Marital status		0.220
Single	53.7	
Married	57.9	
Separated/divorced/widowed	57.4	
Education level		0.515
Certificate	51.1	
Diploma	55.3	
Bachelor’s degree	57.0	
Number of children <6 years		0.562
1	55.2	
≥2	58.1	
Previous injury		**<0.001**
No	51.7	
Yes	91.8	
Tobacco smoking		0.120
Smokers	42.9	
Ex-smokers	52.7	
Never smoked	56.3	
Regular physical exercise (hours per week)		**0.024**
≤5	57.6	
>5	47.2	
School level		0.176
Primary school	57.5	
Junior secondary school	52.8	
Senior secondary school	54.1	
Length of employment (years)		0.307
≤10	53.8	
11-20	56.1	
21-30	60.1	
>30	58.1	
Hours of work per week		**0.002**
40	54.2	
>40	65.5	
Average number of students taught		0.591
≤25	51.4	
26-30	57.0	
31-35	57.9	
36-40	55.0	
>40	55.2	
Extracurricular activities		0.623
No	56.6	
Yes	55.2	

^a^Statistical associations between independent variables and LBP were evaluated using chi-squared. Statistically significant differences (p < 0.05) are marked in bold.

The results suggest that teachers who reported that their job required high physical effort, rapid physical activity, awkward body and awkward arm had a higher prevalence of LBP (Table 4). These findings were statistically significant. The prevalence of LBP was higher among teachers with high psychosocial job demands (57.4%) and high job dissatisfaction (58.6%) when compared to those with low psychosocial job demands (48.9%) and low job dissatisfaction (51.7%), respectively; with a statistical difference of p < 0.05.

**Table 4:** **The 12 month prevalence of LBP among Botswana teachers in relation to physical and psychosocial factors**

Risk factors^a^	% with LBP	***P*** value
Much physical effort		**0.012**
No	51.3	
Yes	58.5	
Lift heavy loads		0.832
No	55.4	
Yes	57.3	
Rapid physical activity		**<0.001**
No	51.2	
Yes	62.2	
Awkward body position		**<0.001**
No	52.2	
Yes	64.6	
Awkward arm position		**<0.001**
No	51.4	
Yes	66.9	
Decision latitude		0.275
No	59.7	
Yes	54.7	
Psychosocial job demands		**0.015**
Low	48.9	
High	57.4	
Job insecurity		0.388
Low	54.9	
High	58.3	
Co-worker support		0.105
Low	60.9	
High	54.7	
Supervisor support		0.394
Low	58.1	
High	54.6	
Social support		0.897
Low	56.9	
High	55.5	
Job dissatisfaction		**0.017**
Low	51.7	
High	58.6	

^a^Statistical associations between independent variables and LBP were evaluated using chi-squared. Statistically significant differences (p<0.05) are marked in bold.

### Risk factors for LBP

As shown in Table 5, the logistic regression model contained ten independent variables. Only six of these independent variables made a unique, statistically significant contribution to the model. The strongest predictor of reporting LBP was previous low back injury, with an adjusted odds ratio of 9.67. Female gender and increasing age were also significantly associated with LBP. Regular physical exercise, with more than 5 hours of exercise per week, remained associated with decreased odds of reporting LBP, compared to those with less hours of physical exercise. Awkward arm position and high psychological job demands also remained associated with LBP in the final, adjusted, model.

**Table 5:** **Risk factors for LBP among Botswana teachers**

Risk factors^a^	Logistic OR(95% CI)	Corrected OR (95% CI)	***P*** value
Gender			
Male	1	1	
Female	1.51 (1.14-2.00)	1.42 (1.12-1.77)	**0.004**
Age (years)			
≤30	1	1	
31-40	1.25 (0.89-1.75)	-	0.203
41-50	1.56 (1.08-2.24)	1.47 (1.07-1.97)	**0.017**
>50	1.46 (0.83-2.55)	-	0.185
Previous injury			
No	1	1	
Yes	9.67 (4.94-18.93)	1.92 (1.74-2.02)	**0.001**
Hours of physical exercise per week (h)			
≤5	1	1	
>5	0.63 (0.43-0.93)	0.64 (0.45-0.93)	**0.021**
Much physical effort			
No	1		
Yes	1.10 (0.81-1.49)	-	0.539
Rapid physical activity			
No	1		
Yes	1.12 (0.82-1.53)	-	0.475
Awkward body position			
No	1		
Yes	1.09 (0.75-1.59)	-	0.649
Awkward arm position			
No	1	1	
Yes	1.81 (1.24-2.62)	1.39 (1.14-1.63)	**0.002**
Psychosocial job demands			
Low	1	1	
High	1.40 (1.02-1.93)	1.34 (1.02-1.76)	**0.040**
Job dissatisfaction			
Low	1		
High	1.23(0.95-1.60)	-	0.119

^a^Risk factors evaluated simultaneously using logistic regression and expressed as Logistic Odds Ratios (OR) with 95% Confidence Intervals (95% CI). All OR adjusted for gender and age.

^b^Odds ratios with statistically significant results corrected using the formula of Zhang & Kai[30]. Statistically significant differences (p<0.05) are marked in bold.

### LBP disability

**Figure 1:**
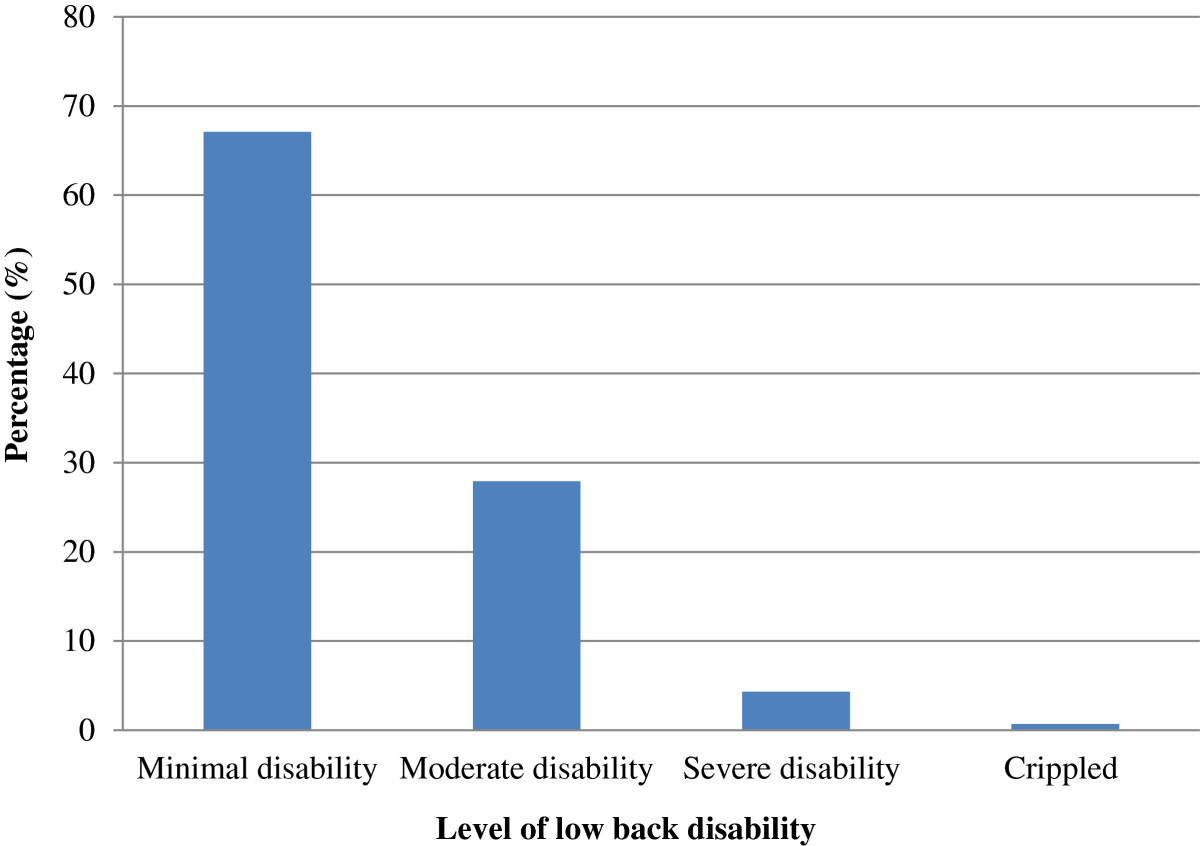
**Level of low back disability among Botswana school teachers with LBP.**

As shown in Figure 1, the majority of teachers with LBP (67.1%) reported minimal disability. Moderate disability was reported by almost a quarter of teachers with LBP (27.9%). Severe disability and being crippled were reported by a relatively low proportion of teachers with LBP; being 4.3% and 0.7%, respectively.

### Risk factors for LBP disability

Various factors were statistically associated with LBP disability during chi-squared tests. Among individual factors, gender, age, body mass index, education level and previous low back injury were significantly associated with LBP disability all with p-values of less than 0.001. Lifestyle factors included tobacco smoking (p = 0.022). Work related factors included the level of school at which teachers taught (p < 0.001) and length of employment (p = 0.001). Refer to Table 6. Chi-squared tests for independence with Yates Continuity Correlation indicated a significant association between LBP disability and physical effort (p < 0.001), lifting heavy loads (p = 0.030), rapid physical activity (p < 0.001), awkward arm (p < 0.001) and awkward arm (p < 0.001) (Table 7). As shown in Table 8, LBP disability was associated with psychosocial job demands, job insecurity and supervisor support. However, not all factors remained statistically significant when evaluated in the logistic regression model. Of all the evaluated variables, only female gender (OR: 2.47, 95% CI: 1.52-3.99, p < 0.001) and previous low back injury (OR: 3.01, 95% CI: 1.92-4.74, p < 0.001) were shown to be significant contributors to LBP disability (Table 9).

**Table 6:** **Individual factors associated with LBP disability among Botswana school teachers**

Risk factors^a^	Minimal disability	Moderate/severe disability/crippled	Total	***P*** value
	%	%	%	
Gender				**<0.001**
Male	27.7	14.5	23.3	
Female	72.3	85.5	76.7	
Age				**<0.001**
≤30	23.5	13.3	20.2	
31-40	38.4	35.3	37.4	
41-50	29.1	40.1	32.7	
>50	9.0	11.3	9.7	
Body mass index				**<0.001**
<18.5	5.6	4.1	5.1	
18-24.9	46.3	30.3	41.1	
25-29.9	27.2	33.0	29.1	
≥30	21.0	32.6	24.7	
Marital status				0.337
Single	52.7	47.9	51.1	
Married	41.7	45.1	42.8	
Separated/divorced/widowed	5.6	6.9	6.0	
Educational level				**<0.001**
Certificate	3.7	7.6	5.0	
Diploma	55.5	64.0	58.3	
Bachelors’ degree	40.8	28.4	36.7	
Number of children less than 6 years				1.000
1	72.3	71.8	72.2	
≥2	27.7	28.2	27.8	
Previous injury				**<0.001**
No	88.3	74.4	83.7	
Yes	11.7	25.6	16.3	
Tobacco smoking				**0.022**
Never smoked	91.0	95.6	92.5	
Ex-smokers	6.2	2.5	5.0	
Current Smokers	2.8	1.9	2.5	
Hours of physical exercise per week (h)				0.700
≤5	88.8	90.1	89.2	
>5	11.2	9.9	10.8	
School level				**<0.001**
Primary school	55.0	69.7	59.9	
Junior secondary	33.2	25.2	30.6	
Senior secondary	11.7	5.0	9.5	
Length of employment (years)				**0.001**
≤10	50.9	38.5	46.8	
11-20	32.0	37.2	33.7	
21-30	15.3	20.2	16.9	
>30	1.9	4.1	2.6	
Working hours per week (hours)				1.000
40	85.0	85.2	85.1	
>40	15.0	14.8	14.9	
Number of children taught				0.060
≤25	12.8	8.2	11.3	
26-30	15.5	14.8	15.2	
31-35	28.3	30.6	29.0	
36-40	32.5	38.5	34.4	
>40	11.0	7.9	10.0	
Extracurricular activities				0.309
No	35.1	33.9	33.9	
Yes	64.9	68.5	66.1	

^a^Statistical associations between independent variables and LBP disability was evaluated using chi-squared. Statistically significant differences (p<0.05) are marked in bold.

**Table 7:** **Physical factors associated with LBP disability among Botswana school teachers**

Risk factors^a^	Minimal disability	Moderate/severe disability/crippled	Total	***P*** value
	%	%	%	
Much physical effort				**<0.001**
No	41.4	26.8	36.6	
Yes	58.6	73.2	63.4	
Lift heavy loads				**0.030**
No	85.4	79.6	83.5	
Yes	14.6	20.4	16.5	
Rapid physical activity				**<0.001**
No	59.9	43.9	54.6	
Yes	40.1	56.1	45.4	
Awkward body position				**<0.001**
No	71.9	58.9	67.6	
Yes	28.1	41.1	32.4	
Awkward arm position				**<0.001**
No	71.2	58.3	66.9	
Yes	28.8	41.7	33.1	

^a^Statistical associations between independent variables and LBP disability were evaluated using chi-squared. Statistically significant differences (p<0.05) are marked in bold.

**Table 8:** **Psychosocial factors associated with LBP disability among Botswana school teachers**

Risk factors^a^	Minimal disability	Moderate/severe disability/crippled	Total	***P*** value
	%	%	%	
Decision latitude				0.541
Low	19.3	17.5	18.7	
High	80.7	82.5	81.3	
Psychosocial job demands				**0.040**
Low	20.3	14.6	18.4	
High	79.7	85.4	81.6	
Job insecurity				**0.010**
Low	76.9	68.8	74.2	
High	23.1	31.2	25.8	
Co-worker support				0.071
Low	17.8	22.9	19.5	
High	82.2	77.1	80.5	
Supervisor support				**0.037**
Low	30.3	37.3	32.6	
High	69.7	62.7	67.4	
Social support				0.128
Low	13.8	17.8	15.2	
High	86.2	82.2	84.8	
Job dissatisfaction				0.069
Low	42.2	35.8	40.1	
High	57.8	64.2	59.9	

^a^Statistical associations between independent variables and LBP disability were evaluated using chi-squared. Statistically significant differences (p<0.05) are marked in bold.

**Table 9:** **Individual, physical and psychosocial factors associated with LBP disability among Botswana school teachers**

Risk factors^a^	Odds ratio (OR)	95% CI confidence intervals (95% CI)	***P*** value
Gender			
Male	1		
Female	2.47	1.52-3.99	**<0.001**
Age (years)			
≤30	1		
31-40	1.39	0.77-2.51	0.280
41-50	1.53	0.74-3.20	0.255
>50	1.03	0.39-2.73	0.954
Body mass index			
<18.5	1		
18.5-24.9	1.19	0.49-2.88	0.707
25.0-29.9	1.63	0.66-4.00	0.291
≥30	1.80	0.72-4.49	0.208
Education level			
Certificate	1		
Diploma	0.88	0.39-2.02	0.769
Bachelor degree	0.53	0.22-1.29	0.160
Previous injury			
No	1		
Yes	3.01	1.92-4.74	**<0.001**
Tobacco smoking			
Never smoked	1		
Ex-smoker	0.36	0.13-1.02	0.054
Current smoker	1.64	0.53-5.09	0.393
School level			
Primary school	1		
Junior secondary	0.99	0.63-1.57	0.974
Senior secondary	0.83	0.38-1.85	0.656
Length of employment (years)			
≥10	1		
11-20	1.20	0.73-1.97	0.484
21-30	1.18	0.59-2.36	0.631
>30	1.22	0.35-4.34	0.755
Much physical effort			
No	1		
Yes	1.31	0.82-2.07	0.256
Lifting heavy loads			
No	1		
Yes	0.93	0.56-1.55	0.776
Rapid physical activity			
No	1		
Yes	1.31	0.85-2.03	0.220
Awkward body position			
No	1		
Yes	1.06	0.65-1.72	0.811
Awkward arm position			
No	1		
Yes	1.57	0.98-2.51	0.062
Psychosocial job demands			
Low	1		
High	1.31	0.79-2.17	0.295
Job insecurity			
Low	1		
High	1.31	0.86-1.98	0.211
Supervisor support			
Low	1		
High	0.74	0.50-1.09	0.123

^a^Risk factors evaluated simultaneously using logistic regression and expressed as Odds Ratios (OR) with 95% Confidence Intervals (95% CI). All OR adjusted for gender and age. Statistically significant differences (p<0.05) are marked in bold.

## Discussion

### LBP prevalence

The first aim of this study was to estimate the 12-month prevalence of LBP among school teachers in Botswana. This study found a 55.7% prevalence of LBP among teachers. Parallels can be drawn to other studies where 53.3% of Filipino[19], 53.8% of Ethiopian[20] teachers and 59.2% of Chinese primary and secondary school teachers[21] reported having LBP. The prevalence of LBP found in this study was relatively lower than those reported in studies conducted among female secondary school Saudi (63.8%)[22], Indian (66.2%)[23], Iranian (71.9%)[24] and Turkish teachers (74.9%)[25]. A relatively high prevalence of LBP, 84.0%, was found among Slovenian physical education teachers in a previous study[8]. The LBP prevalence rate in this study was, however, higher than that reported in another Turkish study (51.4%)[26] and other studies carried out among Chinese, Brazilian and Malaysian teachers with LBP prevalence rates of 45.6%, 41.1% and 40.4%, respectively[10, 11, 19]. Lower LBP prevalence levels have also been reported in studies that were conducted among teachers in Malaysia (40.4%)[27], China (40.0%)[28] and France (34.8)[29]. Lower levels of LBP prevalence were further reported among school teachers in Japan (20.6%)[1] and Estonia (11.8%)[9].

### LBP risk factors

Another aim of this study was to determine risk factors associated with LBP among school teachers in Botswana. Chi-squared tests were initially used to determine basic associations between LBP and risk factors. Logistic regression was used to analyze the association of factors that were positively associated with LBP when using chi-squared tests. Logistic regression analysis revealed a number of interesting correlations between LBP and individual, lifestyle, physical and psychosocial factors. Odds ratios with statistically significant results were further corrected using the formula of Zhang and Kai[30].

### Individual factors

In this study, female teachers reported a significantly higher prevalence of LBP (58.7% vs 47.7%) when compared to their male counterparts. Female teachers were one-and-a-half times more likely to experience LBP (OR: 1.51, 95% CI: 1.14-2.00), which is consistent with some previous studies conducted in the teaching profession[20, 26] and elsewhere[31, 32]. Female teachers appear to consistently report more LBP than their male colleagues. Supporting this hypothesis are the results of a study of self-reported musculoskeletal symptoms among Turkish teachers which found that female teachers were 2.50 times more likely to report back pain when compared to their male counterparts[33]. In addition, Ethiopian female teachers were found to be more than three times likely to develop LBP in comparison to their male colleagues (OR: 3.23, 95% CI: 2.10-5.26)[20]. A similar link has been found between female gender and LBP among school teachers in Brazil (OR: 1.54, 95% CI: 1.22-2.07)[11]. Similar findings were also documented in a study conducted in Iran where more female teachers reported lower back pain (77.0% vs 69.0%) in comparison to their male colleagues[24]. In a Chinese study of school teachers, the percentage of female teachers was higher than that of their male counterparts in reporting LBP (52.6% vs 45.1%, p < 0.01)[21]. Conversely, a study of Filipino teachers did not show any gender differences between teachers with or without LBP[19]. Similar results were found in a study of university staff where gender was not significantly associated with LBP (p = 0.226)[34]. Furthermore, no significant association has been found between gender and LBP (OR: 1.15, 95%CI: 0.77-1.72) among physical education teachers in Slovenia[8].

One possible reason for gender differences in this study could be the nutritional status of female teachers, given that a higher proportion was found to be overweight when compared with their male counterparts. Even though BMI was not significantly associated with LBP in this study, females had a higher average BMI than males (27.6 ± 7.0 vs 24.8 ± 5.8, p < 0.001). Older age and long teaching experience might also be contributing factors, as females were significantly older than males (39.3 ± 9.0 vs 36.3 ± 7.0 years, p < 0.001) and had a significantly longer working experience than their male colleagues (13.4 ± 8.8 vs 10.1 ± 6.3 years, p < 0.001). Another reason could be that male teachers were involved in more regular physical exercise than females (18.1% vs 10.4%, p < 0.001).

The results of this study suggest that increasing age increases the odds of developing LBP. Teachers who were 41–50 years were 1.56 times more likely to report LBP when compared to those who were 30 years or younger. This result is consistent with a study conducted in Brazil in which teachers aged 40 years and above reported having more back pain than their younger colleagues[11]. Parallels could also be drawn to the results of a Turkish study where teachers over the age of 40 years reported having experienced musculoskeletal pain (p < 0.001)[26]. Increasing age has also been positively associated with LBP in another study of Turkish teachers (OR: 1.05, 95% CI: 1.02-1.08)[25]. Similarly, in a study carried out in Ethiopia, teachers who were 40 years and above were 2.34 times more likely to develop LBP while those in the age group of 30 to 40 years were 1.70 times more likely to develop LBP, compared to those who were less than 30 years[20]. In addition, increasing age was found to increase the odds of LBP (OR: 1.05, 95% CI: 1.03-1.07)[8]. It has been suggested that the likely reason for higher prevalence of LBP among older teachers is that, as people age, there is a gradual decline in muscle mass and they lose connective tissue elasticity and undergo a thinning of the cartilage between joints. On the other hand, healing slows down with advancing age while the body is simultaneously dealing with lifetime accumulated soft tissue damage[11, 26, 35].

Logistic regression analysis revealed that prior injury was independently and significantly associated with LBP among Botswana teachers (OR 9.67, 95% CI 4.94-18.93). However, when this logistic odds ratio was corrected teachers who reported prior injury were found to be 1.92 times more likely to report LBP in comparison to those who did not report priory injury (95% CI: 1.74-2.02). This finding was similar to the results of a study conducted in Ethiopia where it was reported that teachers with a history of low back injury were 1.96 times more likely to develop LBP than those who had no history of low back injury (OR: 1.96, 95% CI: 1.04-3.96)[20]. A similar link has been demonstrated between prior injury and upper extremities, back and lower extremities among male steelworkers in Korea[36] and between prior injury and subsequent injury[37]. Previous musculoskeletal clinical history has also been linked with the development of MSD among Italian health care workers[38].

On the other hand, results of this study suggest that regular physical exercise was negatively associated with LBP. Teachers who reported more than 5 hours of physical exercise a week were less likely to report LBP (OR: 0.63, 95% CI: 0.43-0.93), compared to those who exercised less than 5 hours per week. Similar findings have been demonstrated in a study of school teachers in Ethiopia where teachers who have indicated doing regular physical activity were 0.52 times less likely to report low back pain, compared to those who did not engage in regular physical activity (OR: 0.52, 95% CI: 0.34-0.82)[20]. A similar link has also been demonstrated between habitual physical activity as athletic and MSD among Thai university staff[34]. In a study of Estonian athletes, regular physical exercise 6–11 times per month has been associated with a lower prevalence of knee and hip problems, compared to those who exercised less than 6 times per month. On the other hand, a previous study from Australia found that undertaking no exercise was associated with almost five-fold risk of LBP[39].

### Physical and psychosocial factors

Teachers who reported awkward arm positions at work reported the highest prevalence of LBP in the current study, when compared to those who did not adopt awkward arm positions, which is consistent with some previous research[38, 40, 41]. Teachers who had high psychological job demands were 1.40 more times likely to report LBP than those with low psychological job demands. Similarly, teachers who have reported having stress were 4.15 and 2.18 times more likely to experience LBP in the Philippines and Ethiopia, respectively, than those without stress[19, 20]. High psychological job demands have also been positively correlated to development of musculoskeletal disorders among Polish workers[42]. Additionally, poor mental health has been associated with LBP among Malaysian secondary school teachers (OR: 1.11, 95% CI: 1.06-1.15)[27]. High job demands have also been correlated to LBP among female teachers at a school for the handicapped and among male teachers for classrooms for the handicapped in Japan[1]. On the other hand, a previous study conducted in China among teachers found no statistically significant association between high job demands and LBP[43]. Similar findings have been found for a study conducted in Italy[44].

A possible explanation for the association documented in the current study could be because teachers often work in stressful conditions with large classes, with a lack of educational resources and limited reward for their work[11]. Teachers have also been found to face a high amount of stress during teaching and handling young students and their stress level also increases when having to deal with students with emotional and behavioural problems[23]. It has also been suggested that the more psychological demands needed for a particular task, the greater the possibility to develop any kind of musculoskeletal disorder regardless of the anatomical area[45]. Some research from Japan suggests that this may relate to group dynamics, as well as individual factors[46]. Surprisingly, psychosocial factors such as low decision latitude, high job insecurity, low co-worker, low supervisor and low social support, and high job dissatisfaction were not positively associated with development of LBP in the current study.

### LBP disability

Of those teachers who reported LBP, two-thirds (67.1%) reported experiencing minimal disability while 27.9% reported moderate disability, 4.3% severe disability, and 0.7% reported being crippled. The results of this study demonstrated that none of the respondents had been bed ridden or might have exaggerated their level of pain. This may imply that the majority of teachers probably experienced their LBP at a tolerable level. Conversely, in a study of high school teachers in the Philippines, the majority of teachers were found to experience pain at a barely tolerable level. Of those teachers that reported back pain, 14.5% reported minimal disability, 49.4% reported moderate disability, 25.0% reported severe disability, and 6.0% reported being crippled, while 5.0% reported being bed ridden. The results further indicated that 11% of the teachers may have exaggerated their pain level[19]. In Saudi Arabia, a study of female school teachers found that more than half (53.3%) of the teachers with LBP reported suffering from significant/disabling pain, while 25.9% and 20.8% reported non disabling pain and no pain, respectively[22]. In Slovenia, 19.0% of teachers reported experiencing LBP very often, 30.0% often and 34.0% rarely[8]. Moreover, in the US, 55.0% of preschool workers who reported back pain described it as very or extremely uncomfortable[47]. In a study of Turkish hospital staff, only 11.1% reported mild LBP whereas 63.0% reported moderate pain, 23.1% severe pain and 2.7% very severe pain[48]. Although majority of respondents with LBP in the current study reported minimal disability, strategic measures must be put in place to minimise the progression of their disability from minimal to significant disability. These measures should also be aimed at reducing the level of pain for those with moderate/severe disability to minimal disability.

### Risk factors for LBP disability

The results of logistic regression analysis have shown that female gender generally increases the odds for LBP disability among Botswana teachers. Female teachers were 2.47 times more likely to experience moderate/severe disability or being crippled than their male colleagues (OR: 2.47, 95% CI: 1.52-3.99, p < 0.001). The corrected logistic odds ratio showed that female teachers were 2.31 times more likely to report moderate/severe disability or being crippled than male teachers (95% CI: 1.53-3.49). Similar findings have also been found in a study of Turkish teachers where females reported more severe pain than their male counterparts in the upper back (p = 0.008) and lower back (p = 0.022)[26]. Contrary to these results are the findings of a Chinese study that did not find any significant difference in the LBP disability among teachers. That study rather found that female teachers experienced a higher pain intensity in the shoulder than male teachers (p < 0.001)[21].

A history of low back injury was strongly associated with low back disability in the chi-squared and multiple logistic regression analyses of data in the current study. Previous injury at the lower back region was positively associated with LBP disability among teachers who had reported experiencing LBP (OR: 3.01, 95% CI: 1.92-4.74, p < 0.001), with corrected logistic odds ratios 2.02 (95% CI: 1.57-4.47). Parallels can be drawn to the results of a study carried out among high school students from Starr County, Texas, where previous back injury was positively associated with severe back pain (OR: 9.04, 95% CI: 3.55-23.01)[49]. The literature suggests that, although research has been carried out to determine the prevalence and risk factors of LBP among school teachers, little research has been conducted to establish the level of disability caused by these disorders in the teaching profession.

### Limitations

A number of limitations were identified in the current study. As a cross-sectional study, only associations can be established but no inferences of causality can be made. Further limitations of this study that need to be acknowledged are the possibility of recall bias and self-reporting of LBP. It is not clear if participants correctly remembered the presence of LBP in the last 12 months which could lead to over or under estimation. The presence of LBP depends solely upon the subjective self-report of the participants and not based upon an objective clinically verified diagnosis of a specialist. There could also be underestimation of the role of the risk factors assessed due to the large number of independent variables within the logistic regression analysis.

## Conclusions

Overall, this study has shown that LBP is reasonably common among teachers in Botswana and comparable to the prevalence rates documented in other countries. A wide variety of LBP risk factors were identified during logistic regression analysis, suggesting that the aetiology of this condition is complex and multifactorial in nature. Female gender and previous injury were both positively associated with LBP presence and disability. The complex nature of LBP risk factors found in this study suggests than no single specific preventative or intervention strategy will help in reducing these conditions. As such, to help reduce the prevalence, progression and burden of LBP among Botswana teachers, a greater emphasis should now be placed on ergonomics education, regular physical exercise and occupational stress.

## Authors’ original submitted files for images

Below are the links to the authors’ original submitted files for images. Authors’ original file for figure 1
