# Supplementary figures and images for: Low back pain among school teachers in Botswana, prevalence and risk factors
Source: BMC Musculoskelet Disord. 2014 Oct 30;15:359. doi: 10.1186/1471-2474-15-359 (PMC4230345; doi:10.1186/1471-2474-15-359)

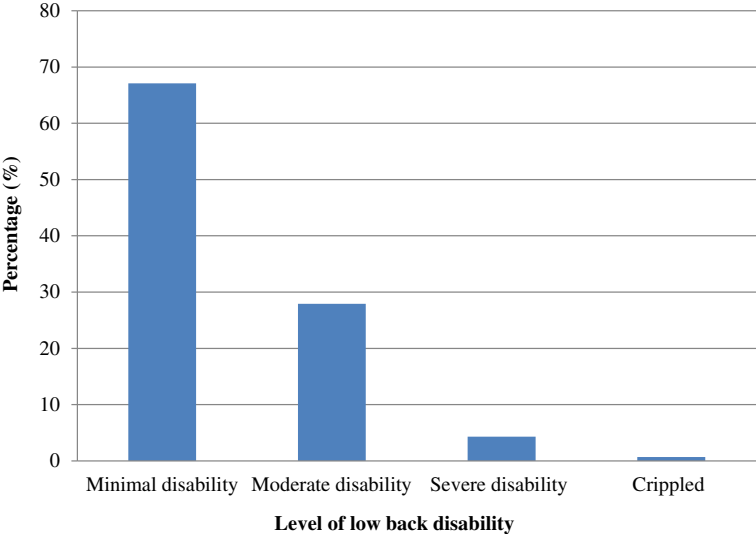

Supplement: Supplementary file 1 — Authors’ original file for figure 1 [file 12891_2014_2300_MOESM1_ESM.pdf]
